# New method for detection of complex 3D fracture motion - Verification of an optical motion analysis system for biomechanical studies

**DOI:** 10.1186/1471-2474-13-33

**Published:** 2012-03-09

**Authors:** Stefan Doebele, Sebastian Siebenlist, Helen Vester, Petra Wolf, Ulrich Hagn, Ulrich Schreiber, Ulrich Stöckle, Martin Lucke

**Affiliations:** 1 Department of Trauma Surgery, Klinikum rechts der Isar, Technische Universität München, Ismaninger Str. 22, 81675 Munich, Germany; 2 Institut of medical statistics and epidemiology, Technische Universität München, Ismaninger Str. 22, 81675 Munich, Germany; 3 Institute of Robotics and Mechatronics, German Aerospace Center, DLR, Oberpfaffenhofen, Münchner Straße 20, 82234 Weßling, Germany; 4 BGU Klinik Tübingen, Schnarrenbergstr. 96, 72076 Tübingen, Germany

## Abstract

**Background:**

Fracture-healing depends on interfragmentary motion. For improved osteosynthesis and fracture-healing, the micromotion between fracture fragments is undergoing intensive research. The detection of 3D micromotions at the fracture gap still presents a challenge for conventional tactile measurement systems. Optical measurement systems may be easier to use than conventional systems, but, as yet, cannot guarantee accuracy. The purpose of this study was to validate the optical measurement system PONTOS 5M for use in biomechanical research, including measurement of micromotion.

**Methods:**

A standardized transverse fracture model was created to detect interfragmentary motions under axial loadings of up to 200 N. Measurements were performed using the optical measurement system and compared with a conventional high-accuracy tactile system consisting of 3 standard digital dial indicators (1 μm resolution; 5 μm error limit).

**Results:**

We found that the deviation in the mean average motion detection between the systems was at most 5.3 μm, indicating that detection of micromotion was possible with the optical measurement system. Furthermore, we could show two considerable advantages while using the optical measurement system. Only with the optical system interfragmentary motion could be analyzed directly at the fracture gap. Furthermore, the calibration of the optical system could be performed faster, safer and easier than that of the tactile system.

**Conclusion:**

The PONTOS 5 M optical measurement system appears to be a favorable alternative to previously used tactile measurement systems for biomechanical applications. Easy handling, combined with a high accuracy for 3D detection of micromotions (≤ 5 μm), suggests the likelihood of high user acceptance. This study was performed in the context of the deployment of a new implant (dynamic locking screw; Synthes, Oberdorf, Switzerland).

## Background

Various conditions are important for sufficient fracture-healing. In addition to adequate blood supply and a reduction in fracture size, axial interfragmentary motion is one of the most important factors for indirect (secondary) bone healing [1-6]. A deficiency in callus formation and delayed or non-union have been reported in diverse studies as the result of inadequate interfragmentary movements [1,7-11]. The optimal range for this micromotion seems to be 400 μm [8]. Therefore, current biomechanical analyses have focused on the development of osteosynthetic implants for optimal interfragmentary motion [1,2,9,12]. In the context of the development of new implants biomechanical tests are highly important. The purpose of this study was to find a motion analysis system for biomechanical tests, which allows the detection of three-dimensional interfragmentary motion with a high accuracy directly at the fracture gap of biomechanical specimens (osteosyntheses). Conventional tactile measurement systems are highly accurate (up to1 μm), but the test is time-consuming, laborious, and at times defective. A further disadvantage of tactile systems is the capture of movement direction in only 1 dimension per tactile unit in most cases. The integration of tactile measurement systems in an existing biomechanical set up could be exceedingly difficult. The integration of optical measurement systems in established biomechanical set ups is easy [13]. Using optical systems there is no interaction between test set up and measurement system. The detection of three-dimensional motion is also possible. But is it possible to detect interfragmentary motion in a range of about 400 μm [8,14]? The optical measurement system PONTOS 5 M (GOM - Optical Measuring Techniques, Braunschweig, Germany) is an established system for motion analysis in the automotive and aerospace industry (used for crash-tests and vibration-analysis of airplane wings). In this study we evaluate PONTOS 5 M for the detection of 3D interfragmentary micromotion in a standardized fracture model. For reference, we used a tactile measurement system consisting of 3 dial indicators designed at the German aerospace centre DLR (Oberpfaffenhofen, Germany). We hypothesized that the optical measurement system would provide the same or higher accuracy for detecting fracture gap movements as the conventional system.

## Results

### Indirect measurement of the fracture movement

**Figure 1:**
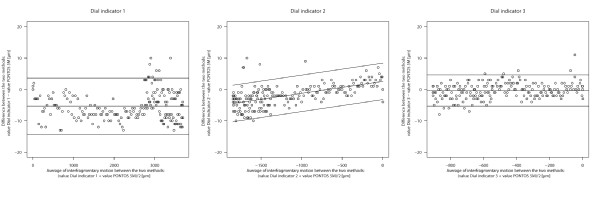
**Bland Altman Plot: Direct comparison of both Systemes**. The differences of motion between the dial indicators and PONOTS 5 M are plotted against the average of the methods. The solid line indicates the systematic bias between the methods. The upper and lower limits show the 95%limits of agreement.

Figure 1 shows the agreement between the three dial indicators and PONOTS 5 M. For dial indicators 1 and 3 there was no obvious dependence of the amount of measuring spindle motion and the mean differences between the direct (PONTOS 5 M) and indirect measurement (dial indicator). For dial indicator 1 there was a bias of -5.3 μm with limits of agreement of -14.3 μm and 3.6 μm. For dial indicator 3 the mean difference was 0.4 μm with limits of agreement of -5.3 μm and 4.6 μm. Dial indicator 2 showed a slight tendency for dependency of mean difference and size of the measurement. The regression approach in Figure 1 indicates this relationship. There is a bias, which tends to be zero for small interfragmentary motion and is the greater the greater the motion is: with an average motion of 0 μm the bias was 2.5 μm, but at an average of -1.8 μm the bias was -4.5 μm. The 95% limits of agreement were of 5 μm around the bias.

### Direct measurement of the fracture movement

**Figure 2:**
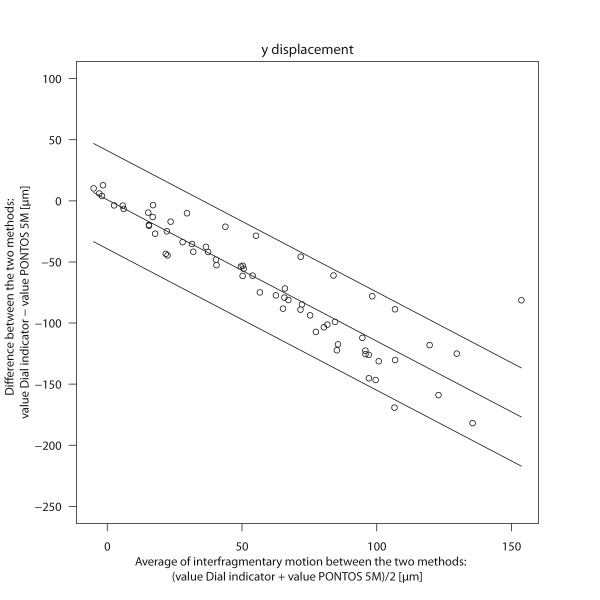
**Bland Altman Plot: Interfragmentary motion Δy**. The differences of interfragmentary motion (Δy) between the dial indicator and PONOTS 5 M are plotted against the average of the two methods. The solid line indicates the systematic bias between the two methods. The upper and lower limits show the 95% limits of agreement.

Regarding the y-displacement of the interfragmentary motion there was a clear dependency of the magnitude of the motion and the difference between the dial indicator and PONTOS 5 M: the higher the load the higher the differences between the two methods (Figure 2). For an average interfragmentary motion of 0 mm the bias between the dial indicator and PONTOS 5 M was 2 μm. For an averaged motion of 120 μm in contrast, the bias was -140 μm. The limits of agreement were of 40 μm around the bias (Table 1).

**Table 1:** Mean differences and 95% limits of agreement

Force[N]	Δy [μm]	Δ*z *[μm]	α [deg]
0	-5 [-45; 35]	-37 [-77; 3]	-0.05 [-0.11;0.01]

10	-7 [-48; 33]	-32 [-80; 16]	-0.04 [-0.13; 0.05]

20	-21 [-61; 19]	-27 [-83; 29]	-0.02 [-0.15; 0.10]

30	-37 [-77; 3]	-21 [-86; 44]	-0.01 [-0.17; 0.16]

40	-52 [-92; -12]	-15 [-89; 59]	0.01 [-0.20; 0.22]

50	-66 [-106; -25]	-9 [-93; 75]	0.03 [-0.23; 0.28]

60	-81 [-121; -41]	-2 [-97; 93]	0.04 [-0.26; 0.34]

70	-93 [-134; -53]	5 [-100; 111]	0.06 [-0.29; 0.42]

80	-106 [-146; -65]	13 [-105; 131]	0.09 [-0.32; 0.49]

90	-122 [-163; -82]	21 [-109; 150]	0.11 [-0.35; 0.57]

100	-138 [-179; -98]	27 [-112; 167]	0.13 [-0.38; 0.64]

**Figure 3:**
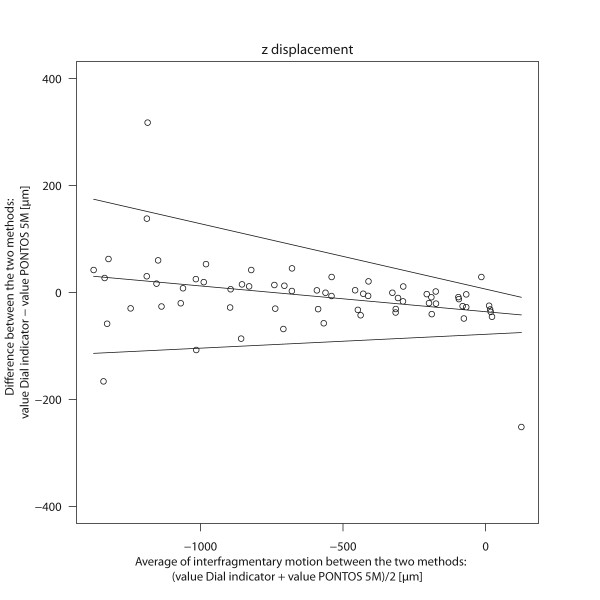
**Bland Altman Plot: Interfragmentary motion Δz**. The differences of interfragmentary motion (Δz) between the dial indicator and PONOTS 5 M are plotted against the average of the two methods. The solid line indicates the systematic bias between the two methods. The upper and lower limits show the 95% limits of agreement.

The agreement of PONTOS 5 M with the dial indicator concerning the z-displacement of the interfragmentary motion is shown in Figure 3. There was a wider spread of the differences with a higher force and therefore a larger interfragmentary motion. For an average interfragmentary motion of -1.31 mm the bias was 30 μm with limits of agreement of -110 and 170 μm. For an average of 0 mm interfragmentary motion the bias was -40 μm with limits of agreement of -80 μm and 10 μm (Table 1).

**Figure 4:**
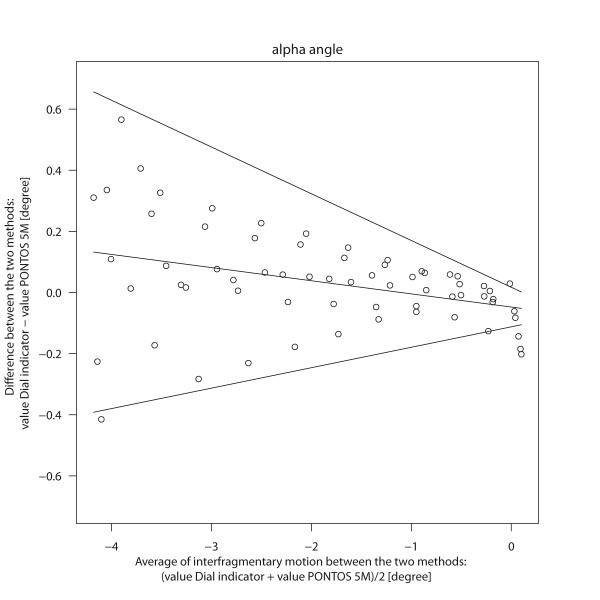
**Bland Altman Plot: Interfragmentary motion Δα**. The differences of interfragmentary motion (α angle) between the dial indicator and PONOTS 5 M are plotted against the average of the two methods. The solid line indicates the systematic bias between the two methods. The upper and lower limits show the 95% limits of agreement.

Regarding the α angle (pitch-angle) there was a bias between the dial indicator and PONTOS 5 M between -0.05 and 0.13 degrees. The difference and especially the variability of the difference clearly depend on the applied load (Figure 4). With a force of 100 N the average angle is -4.1 degree. The bias of the two methods at 100 N is 0.13 degree with limits of agreement of -0.38 and 0.64 degree. For an applied force of 0 N the bias between the methods is -0.05 degree with limits of agreement of -0.11 and 0.01 degree (Table 1).

## Discussion

The aim of the present study was to validate the efficacy of the optical measurement system PONTOS 5 M compared with a conventional tactile measurement system. The results show that both measurement systems were capable of analyzing interfragmentary movements with high accuracy (resolution of about ≤5 μm). However, the optical measurement system was able to analyze 3D motions whereas the tactile system used in this study only performed 2D measurements of the fracture motion. For this reason it was only possible to compare the 2D data obtained using the optical measurement system with the corresponding data of the tactile measurement system. This is one limitation of the present study.

**Figure 5:**
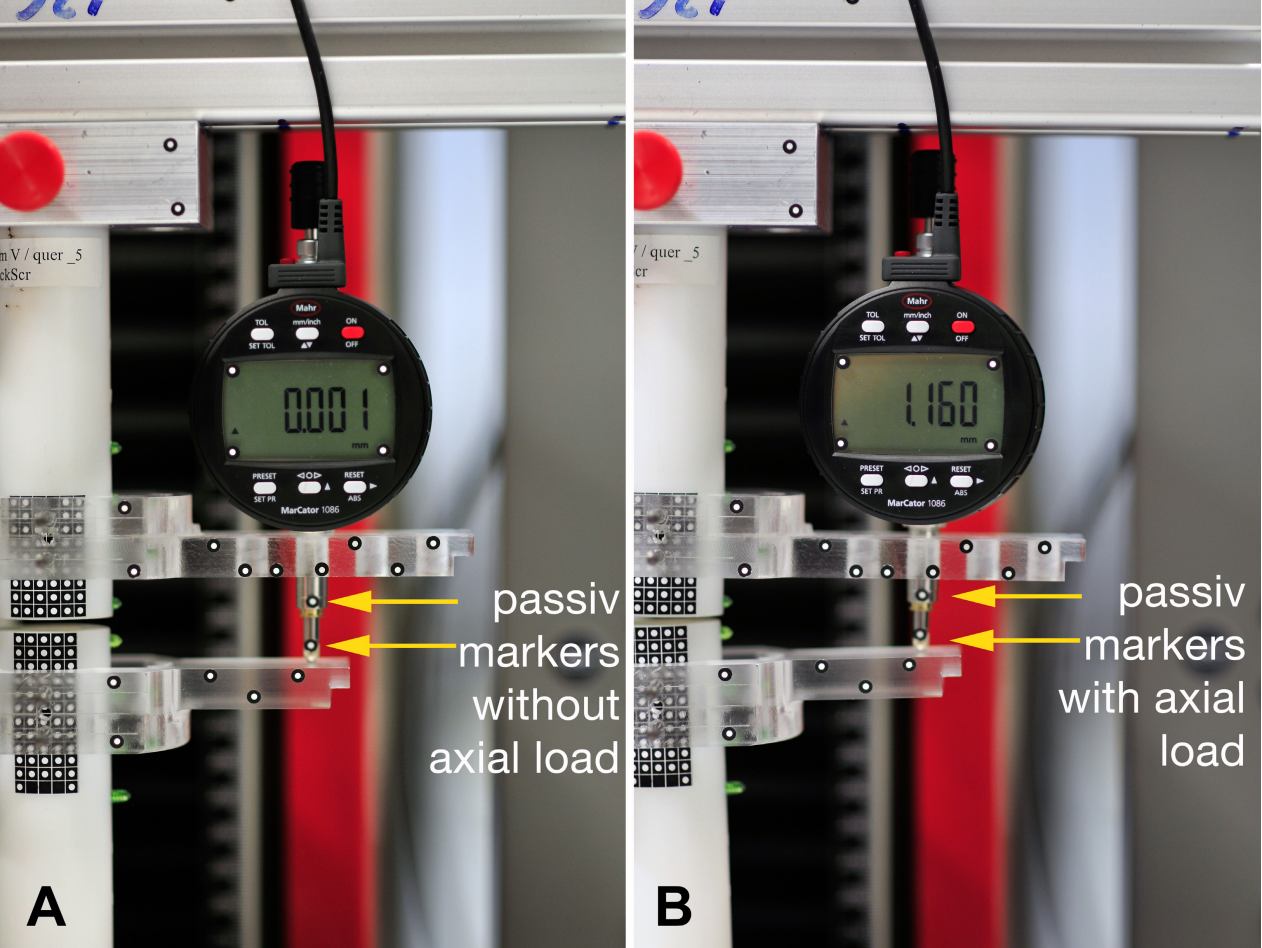
**Additional measurement: Direct comparison of both Systemes**. For this purpose, the passive markers of the optical measurement system were attached to the spindles of the 3 dial indicators.

In order to assess the accuracy of the optical measurement system we attached passive markers of the optical measurement system to the spindle of the dial indicators, analysed the collapse of the measuring spindle (Figure 5) and compared the values of the dial indicators with those of the optical measurement system.

**Figure 6:**
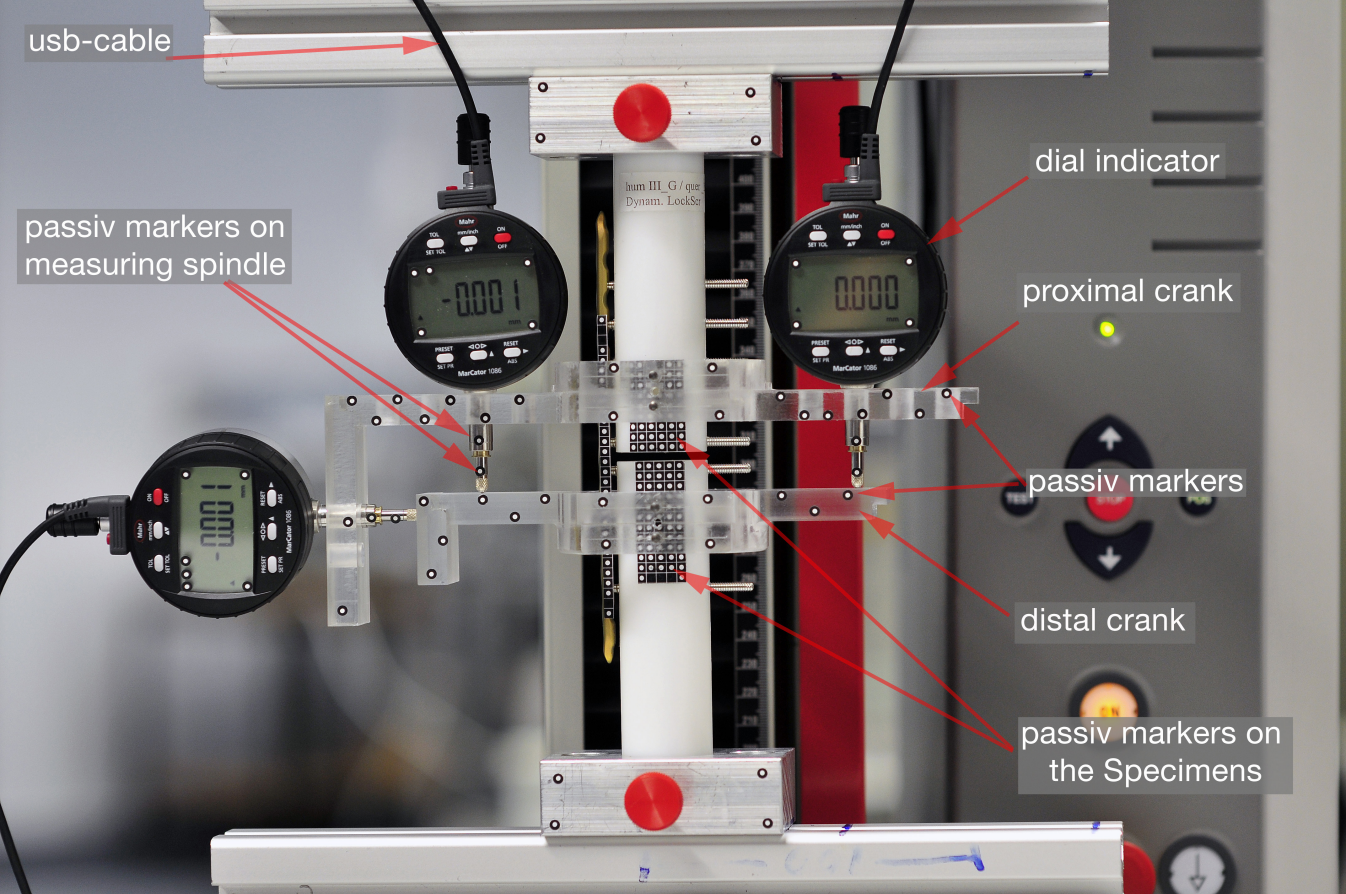
**Test set-up**. Two cranks were fixed to position the 3 dial indicators. Passive markers were fixed directly to the fracture gap.

The resolution of the dial indicators is known to be 1 μm. The accuracy between the tactile measurement system and optical measurement system showed a mean difference of -5.3 μm (dial indicator 1), 0.4 μm (dial indicator 2) and -1.8 μm (dial indicator 3). The variability of the differences between the two methods was between 5 μm and 9 μm. This was only little wider than the error limit of the dial indicators. The difference between the two methods was comparable with the precision within the dial indicators. With the optical measurement system interfragmentary movement can be detected directly at the fracture gap as the passive markers are very small and can be attached nearly anywhere. Contrariwise using the digital indicators the measurement of the interfragmentary motion was only possible indirectly by using the cranks. The average deviation in interfragmentary motion measured directly at the fracture gap (PONTOS) and indirectly using the tactile system and the cranks was 120 μm (y-displacement), -1310 μm (z-displacement) and -4.1 degree (α angle). These results are rather surprising. The large difference of the measured interfragmentary motion can be explained by the set up of the tactile system using the cranks. The cranks become deformed by the mechanical load during the test which was shown by attaching the passive markers on the cranks (Figure 6). Using the cranks tends to result in a decreasing of accuracy. This effect was more pronounced the higher the axial load was.

In addition to the high accuracy of the optical measurement system, this system is much easier to use in comparison to the dial indicator method, because the passive markers are self-adhesive and, as mentioned, can be attached nearly anywhere. The dial indicators, in contrast, each requires a specially produced crank for attachment. This setup is not only time-consuming, but also expensive, and the accuracy of the measurement depends directly on the cranks. In contrast, each passive marker of the optical measurement system functions like a 6-DOF-sensor, so that several different data sets for the object can be obtained. Due to its high accuracy, PONTOS 5 M is regularly used in the automobile and airplane industry for car crash-tests or vibration-analysis of airplane wings. Therefore, large amounts of data are available from different testing setups. In biomechanical setups for musculoskeletal research, diverse types of fracture models have been validated. We decided to use a simple model with a transverse fracture gap in our study in order to exclude measurement deviations as far as possible. There are some publications that deal with the application of optical measuring systems in biomechanics. Here also the accuracy of the systems was part of the research. A common system in the biomechanical field is the Vicon system [15,16]. A study by Windolf et al showed an accuracy of 64 ± 5 microns using the Vicon-460 system [16]. Arbitrary changes in camera arrangement revealed variations in mean accuracy between 76 and 129 μm. This is less accurate than measuring with the PONTOS System.

## Conclusions

In this study, we attempted to expand the applicability of the PONTOS 5 M optical measurement system for biomechanical assessments. As the need for implant improvement, especially with regard to plate stiffness, is acute, the need for an accurate, validated, easy-to-handle 3D measurement system is also high. Within the framework of the presented data and the limitation of only 2D evaluation, we can say that the PONTOS 5 M optical measurement system appears to be a favourable alternative to previously used tactile measurement systems for biomechanical applications. Easy handling combined with a high accuracy (≤ 5 μm) and 3D detection of motions suggests the likelihood of high user acceptance. The use of simple passive markers suggest an easier handling at cadaver models as compared to mechanical devices, which need accurate disinfection after usage.

## Methods

**Figure 7:**
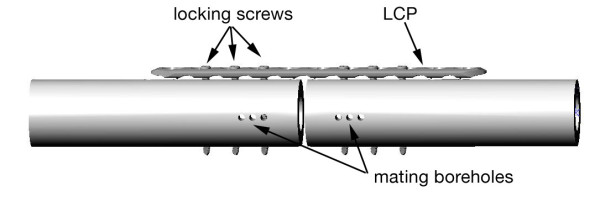
**Osteosynthesis. Osteosynthesis with standard 11-hole, 3.5-mm locking compression plate (Synthes, Oberdorf, Switzerland)**. Plates were fixed with 3 locking screws (55 mm) on each fragment placed in the second, third, and fourth hole from the fracture site.

In 6 surrogate specimens, a standardized transverse fracture model with a fracture gap of 3 mm was created, as described previously [1,2]. The cylindrical bone surrogates were manufactured using polyoxymethylen-copolymerisat with a Young's modulus of 3.1 *GPa *(diameter 30 mm, wall thickness 7 mm, cylinder length 120 mm). Osteosynthesis was performed using a bridge-plating configuration with a standard 11-hole, 3.5-mm locking compression plate (Synthes, Oberdorf, Switzerland). Plates were fixed with 3 locking screws (55 mm) on each fragment placed in the second, third, and fourth hole from the fracture site (Figure 7). All screws were tightened to 1.5 Nm, with the plate elevated 2 mm from the surrogate surface. Specimens were inserted into a special testing frame mounted on a testing machine (Zwick 2.5 KN; Ulm, Germany) and an axial load of up to 100 N with a constant rate of 10 mm/min was applied. Measurements of the micromotion of the fragments were performed using a self-made high-accuracy tactile measurement system made of 3 digital dial indicators and an optical measurement system.

### Optical measurement system

**Figure 8:**
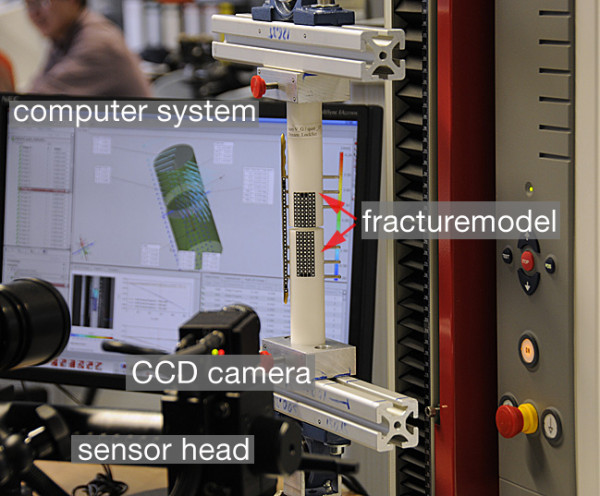
**PONTOS 5 M**. Measuring with the optical motion analysis system PONTOS 5 M.

For measuring the 3D fracture motion we used the optical analysis system PONTOS 5 M (GOM - Optical Measuring Techniques, Braunschweig, Germany). The system is offered in 4 different configurations (5 M, 4 M, 12 M or High Speed) with several camera resolutions (up to 4096 × 3072 pixel) and frame rates (up to 5000 Hz) to cope for diverse applications. The individual system consists of two CCD cameras. For the detection of the motion passive markers are required. These passive markers are simple white dots. The size is adapted to the object size and camera resolution. The points are recorded and tracked by the PONTOS software (Figure 8). The accuracy of the measurements is directly addicted by the resolution of the cameras. Each passive marker is detected in the single image as ellipses in the size of several pixels. The center of the marker can therefore be determined in the sub-pixel space by evaluation of a best fit at the contour of the ellipses, which is done automatically by the software. The PONTOS 5 M system was set up and calibrated for a measurement volume of 350 × 280 × 280 mm according to the manufacturers documentation. The geometrical setup as well as the optical distorsion factors of lenses are considered in the calibration procedure. The frame rate was 4 Hz. We used white self-adhesive dots with a diameter of 2 mm. At least 3 points per object are needed. For a higher accuracy we used all in all about 200 passive markers. It is not necessary to add the points in a special pattern. But doing that, the system could learn to identify the objects with the help of the different patterns. A group of points functions like a six degrees of freedom (6DoF)-sensor. The high number of points allowed fitting two cylindrical geometry elements which represent the physical cylindrical fracture fragments. The 6DoF motion could therefore be directly represented at the location in the centre of the fracture gap for each fragment. Relative motion in all six degrees of freedom where analysedrespectively.

### Reference measurement system

For reference measurements we used digital indicators from the company MAHR (Marcator 1086; Mahr, Goettingen, Germany). To date, dial indicators represent the gold standard in measuring motion in the range of micrometers. We used 3 digital indicators. Each dial gauge can specify one degree of freedom. The resolution of the individual gauge was 1 μm (Span of error 5 μm, Repeatability 2 μm). This accuracy is ensured by the company. For positioning the 3 dial indicators to the osteosynthesis two cranks were constructed and made for measure using a cnc milling machine (Figure 6). The measurement set up was developed in cooperation with the German Aerospace Center (DLR, Oberpfaffenhofen, Germany). The dial indicators were connected to a computer system by an usb interface. The data recording was carried out using the supplied software from Mahr. Due to the placement of the indicators far from the fracture gap, the interfragmentary motion could not be detected directly. Instead, interfragmentary motions were indirectly measured by analyzing the motion of the measuring spindle of the dial indicators. The motion at the fracture gap has to be calculated using the geometry of the cranks and the values of the dial indicators. Using the values of digital indicator 1 and 2 it was possible to calculate the z deviation (Δ*z*) and the angle alpha (Δ*α*) between the both cylinder axis (fracture fragments) (Figure 6). Using digital indicator 3 it was possible to calculate the y deviation (Δy). To compare the accuracy of the two measurement systems (PONTOS 5 M vs tactile measurement system), we conducted an additional measurement. For this purpose, the passive markers of the optical measurement system were attached directly to the spindles of each of the 3 indicators (Figure 5). With this set up the motion of the spindles could be detected with the optical measurement system, by measuring the point to point motion. Both values (digital indicator and PONTOS) could be compared. Simultaneous data collection was achieved by trigger points. A total of 3 data-sets, 200 values per set, were collected, 1 for each dial indicator.

### Statistical analysis

For analyzing the agreement between the optical measurement system PONTOS 5 M and the indirect measuring by the dial indicators Bland-Altman-Plots were calculated. For each comparison we checked the assumption of uniform differences and uniform variability. If these assumptions were violated a regression approach was applied. We used a Generalized Estimation Equation Model (GEE) to account for the different measurements made in one specimen (the force was varied between 0 and 100 N in increments of 10 N for each specimen, which results in 11 measurements per specimen). The results of this model were used to calculate mean differences and 95% limits of agreement as described by Bland and Altman [17,18].

## Competing interests

All authors confirm that they have no financial or non-financial competing interests.

## Authors' contributions

SD designed the study, collected the data, analyzed the data and drafted the manuscript. SS analyzed the data, ensured the accuracy of the data and analysis and drafted the manuscript. HV analyzed the data and approved the final manuscript. PW participated in the design of the study and performed the statistical analysis, ensured the accuracy of the data and analysis. UH designed the study. USch designed the study, analyzed the data and drafted the manuscript. USt participated in design and coordination of the study. ML participated in the design of the study and drafted the manuscript, analyzed the data. All authors read and approved the final manuscript.

## Pre-publication history

The pre-publication history for this paper can be accessed here:

http://www.biomedcentral.com/1471-2474/13/33/prepub
